# An Experimental Study to Replace the Thoracic Descending Aorta for Pigs with a Self-Made Sutureless Blood Vessel

**DOI:** 10.1155/2014/587393

**Published:** 2014-02-18

**Authors:** Fenglin Song, Wenwu Zhou, Tao Tang, Xiaobing Li, Xiaoming Wu, Jinfu Yang

**Affiliations:** ^1^Department of the Cardiovascular Surgery of the 2nd Xiangya Hospital, Central South University, Middle Renmin Road 139, Changsha 410011, China; ^2^Department of the Cardiovascular Surgery of the Hunan Provincial People's Hospital, Middle Jiefang Road 61, Changsha 410005, China

## Abstract

To simplify the procedure of blood vessel replacement operation and shorten the vascular anastomosis time, we developed a special artificial blood vessel which can be connected to native blood vessels without suture. The self-made sutureless blood vessel (SMSBV) was made from two titanium connectors and a Gore-Tex graft. To investigate blood compatibility and histocompatibility of the SMSBV, we carried thoracic descending aorta replacement using either SMSBV or Gore-Tex, respectively, in pigs. The aortic clamp time and the operative blood loss in the experimental group (using SMSBV) were less than those in the control group (using Gore-Tex). The whole blood hematocrit, platelet count, plasma soluble P-selectin, plasma free hemoglobin, and interleukins 2, 6 at each time point were not different significantly between the two groups. Light microscopy and transmission electron microscopy examination showed there were layers of vascular smooth muscle cells and endothelial cells adhered in the inner wall of artificial blood vessel without any signs of thrombosis. Based on the result, we have drawn the conclusion that the application of SMSBV can significantly shorten the vascular anastomosis time, reduce operative blood loss, and show good blood and tissue compatibility.

## 1. Introduction

The surgical treatment for great artery diseases such as aortic aneurysm and aortic dissection remains a challenge for any cardiac surgeon. Because of the time-consuming, hand stitched anastomosis of the graft blood and the native vessels, bleeding during surgery is often difficult to control and anastomotic bleeding is the most frequent complication in operations for aortic dissection [1, 2]. Additionally, the likelihood of hypoperfusion occurring is directly proportional to the operating time, which has been associated with the occurrence of the central nervous system complications [3]. Therefore, reducing the operating time and simplifying the procedure of anastomosis without compromising the operative accuracy have become a key focus of research in the field of vascular surgery. Based on this background, we have developed a special artificial blood vessel, termed the self-made sutureless blood vessel (SMSBV), which can be connected to a native blood vessel without the need for suture. Using the SMSBV, we conducted a series of thoracic descending aorta replacement operations in pigs.

## 2. Materials and Methods

### 2.1. Construction of the SMSBV

**Figure 1:**
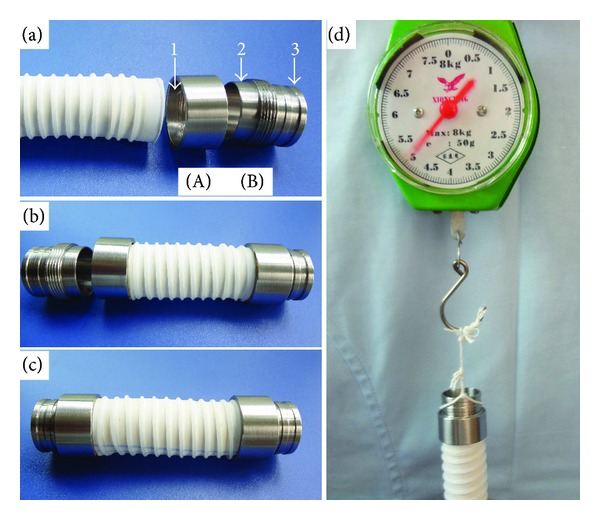
The connection between the self-designed metal connector and Gore-Tex graft. Panel (a) shows the shape of the outer tube (A) and of the inner tube (B); the smooth surfaces of the inner tube and the outer tube are marked with arrow 2 and arrow 1, respectively, and the convex ridge is marked with arrow 3. Panel (b) shows the Gore-Tex graft inserted into the outer tube and panel (c) shows the inner tube screwed into the outer tube, and the inserted Gore-Tex has been trapped into the space between the two smooth surfaces; panel (d) shows that the trapped Gore-Tex was not dislocated when the weight increased to 5 Kg.

This SMSBV was composed of a Gore-Tex artificial vessel (purchased from Gore company, USA) and self-designed metal connectors at both ends of the Gore-Tex. Although this artificial vessel can be made in a variety of different in length and diameter based on different application specifications, the size in this study was 80 mm in length and 12 mm in diameter. The self-designed metal connector was made from titanium alloy (purchased from Shanghai Shape Memory Alloy Material Co., Ltd.) by Changsha Instrument Factory. The metal connector was composed of two parts of the outer tube and the inner tube. The outer tube (Figure 1(a)-(A)) was a short screw pipe with a diameter of 12 mm, a length of 10 mm, and a thickness of 0.5 mm. The screw thread was cast interiorly on the half part of the inner wall (5 mm in length), and the remaining inner wall was kept as a smooth surface (Figure 1(a), arrow 1). The inner tube (Figure 1(a)-(B)) was also a screw pipe with a diameter of 11 mm and a length of 20 mm, and the screw thread was cast exteriorly on the outer wall. The inner tube can be divided equally into two parts: one part was designed as to screw into the outer tube and the other part was to insert into the native artery, and half of the screw part (5 mm in length) was not cast with screw thread (Figure 1(a), arrow 2), and the outer wall of the insert part was cast with two convex ridges (Figure 1(a), arrow 3). The convex ridges (height 0.2 mm, width 1.5 mm, and space between 2 mm) were designed to facilitate the ligation of the native blood vessel onto the insert part. The Gore-Tex graft was cut into a segment of 50 mm length, and 5 mm was inserted into the outer tube of the metal connector (Figure 1(b)), then the inner tube of the metal connector was screwed into the outer tube (Figure 1(c)). Once screwed together, the Gore-Tex graft (5 mm) was trapped by the two smooth surfaces of the inner tube and the outer tube. So the total length of the SMSBV was 80 mm. Tested by spring tension measuring instrument, the trapped Gore-Tex pipeline can withstand the pull of 5 Kg without any *dislocation* (Figure 1(d)). And tested by water pressure test, when the SMSBV lumen endotension increased to 40 Kpa, the artificial vessel did not leak any liquid.

### 2.2. Animal Experiments

#### 2.2.1. Artificial Blood Vessel Implantation

**Figure 2:**
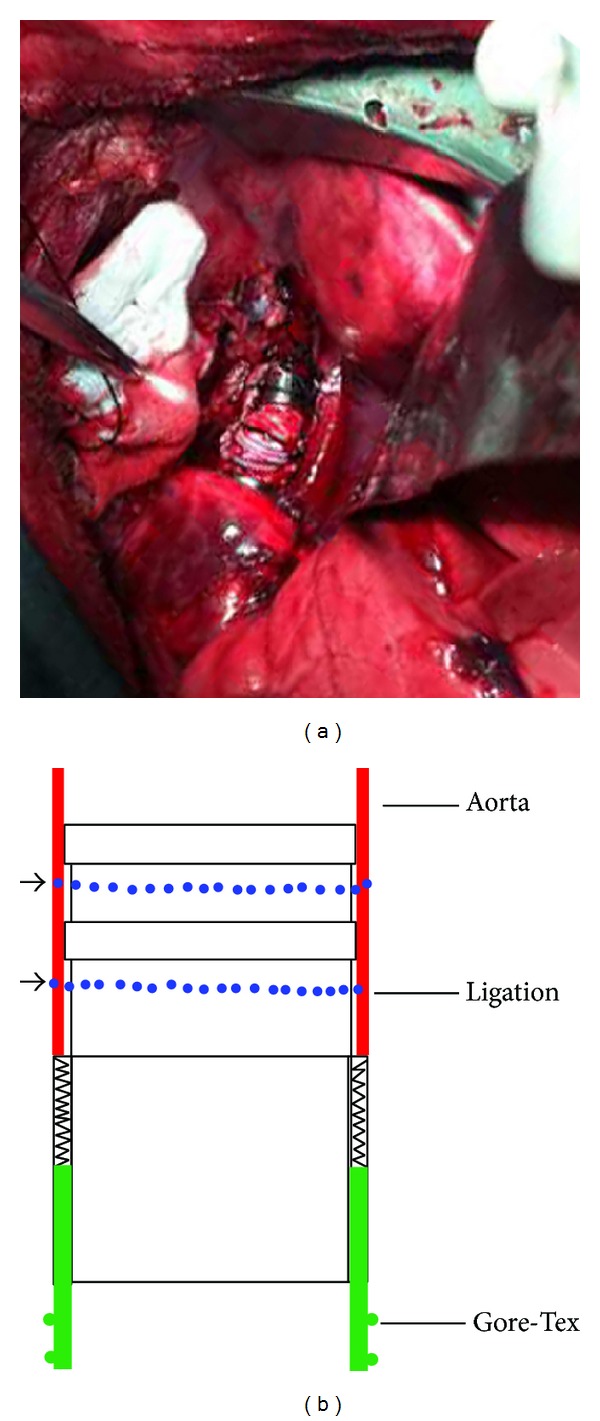
Implantation of SMSBV and its schematic picture. Panel (a) shows that the metal connectors of sutureless vessel have been inserted into the distal and proximal lumens of the thoracic descending aorta, and the sleeved aortic wall was double ligated; panel (b) is a schematic which shows the aortic wall with red color, the Gore-Tex with green color, and the ligature with blue dashed line.

15 pigs (aged 4-5 months and weighing 50–60 kilograms) were divided into the experimental group (7 cases) and the control group (8 cases). After 12 hours of routine preoperative fasting, the anesthesia was started with induction of thiopental intramuscular injection (dosage 1.5 mg/kg). Venous line was established via ear vein and muscle relaxant was used intravenously. After intubation and ventilation, the anesthesia was maintained by intravenous injection of propofol (dosage 8 mg/kg). The thoracotomy was carried out in the left fifth intercostal space and heparin was used with a dosage of 1 mg/kg. To expose and mobilize the thoracic descending aorta with a length of 80 mm to 90 mm, the mediastinal pleura was opened and 2-3 pairs of intercostal arteries were ligated and cut. When the thoracic descending aorta was free, 20 mm and 100 mm distal away from the arterial ligament were cross-clamped, respectively, and between the two clamps, a segment of thoracic descending aorta with a length of 60 mm was cut and removed. For the experimental group, the SMSBV was implanted to the size of removed thoracic descending aorta with a simple maneuver (Figure 2): the metal connectors at the two ends of the artificial vessel were inserted into the distal and proximal lumens of the remaining descending aorta, and the sleeved aortic wall was double ligated to the annular grooves between the convex ridges on connector (Figure 2(b), blue dashed line). When replacing, attention must be paid to not distort the artificial or native vessels. For the control group, the aorta was mobilized and removed almost in the same way as for the experimental group (before the administration of the cross-clamping, a temporary bypass for the descending thoracic aorta was necessary), and then, a segment of Gore-Tex graft with a length of 60 mm and a diameter of 12 mm was implanted with two end-to-end anastomoses by a running suture. Then, protamine was used with a dosage of 1 mg/kg to neutralize the anticoagulation effect of heparin, and after the setting of thoracic drainage tube, the chest was closed. The animals were allowed to awake and weaned from the ventilator and then returned to their cages.

#### 2.2.2. Postoperative Management

After the operation, the swine were fed separately to prevent them from biting one another, and only half of the required food was provided to prevent swine growing too fast, and 3 days after the operation, warfarin (2.5 mg/d) was added into the food for the purpose of anticoagulation. Intramuscular injection of penicillin 800,000 u was used daily till the chest drainage tube was removed 2 days later, and the operative wound was washed with iodine twice a day until the wound stitches were removed 10 days later. Venous blood was sampled at 7 time points (preoperation and postoperation: 30 minutes, 7 days, 15 days, 30 days, 90 days, and 180 days) to check the blood hematocrit (HCT), platelet count, plasma soluble P-selectin, plasma free hemoglobin, and interleukins 2, 6 (IL-2, IL-6) for blood compatibility testing. After the last time of venous blood sampling at 180 days after the operation, all animals were sacrificed and the thoracic descending aorta specimens were collected for light microscopy and transmission electron microscopy (TEM).

### 2.3. Examination of Blood and Tissue Compatibility

The blood HCT and platelet count were checked at the clinical laboratory of the 2nd Xiangya Hospital. The plasma soluble P-selectin, plasma free hemoglobin, and IL-2 and IL-6 were checked by enzyme-linked immunosorbent assay (ELISA) testing at the advanced laboratory of Hunan Ministry of Education.

The thoracic descending aorta specimens were divided into two copies: one for microscopic analysis by the Department of Pathology in the 2nd Xiangya Hospital and the other for transmission electron microscopy (Scan Electronic Microscopy, SEM, Quanta200, FEI) by Xiangya Medical College of Central South University.

### 2.4. Statistical Methods

Data were expressed as mean ± SE. Statistical Product and Service Solutions 13.0 software (SPSS Institute) was used for all analyses. An independent-sample *t*-test was performed to check for differences in each variable at the same time points between the two groups. The critical alpha level for these analyses was set at *P* = 0.05.

## 3. Results

### 3.1. Surgical Results

All the animals recovered from the operation uneventfully and no postoperative spinal cord ischemia-induced paralysis symptoms were found. There were 7 swine in the experimental group undergoing thoracic descending aorta replacement with SMSBV; the aortic clamp time was 10 to 13 (mean 11.2 ± 1.7) minutes and the operative blood loss was 90 to 150 (mean 120 ± 27.8) mL. The 8 swine in the control group underwent thoracic descending aorta replacement with Gore-Tex, and the aortic clamp time was 40 to 66 (mean 52.6 ± 8.4) minutes and the operative blood loss was 300 to 450 (mean 320 ± 41.3) mL. All the animals were fed for 180 days after operation and displayed normal signs of growth. When sacrificed, the pigs' body weight had increased to 100–122 (mean 109 ± 8.3) kilograms.

### 3.2. General Observations

**Figure 3:**
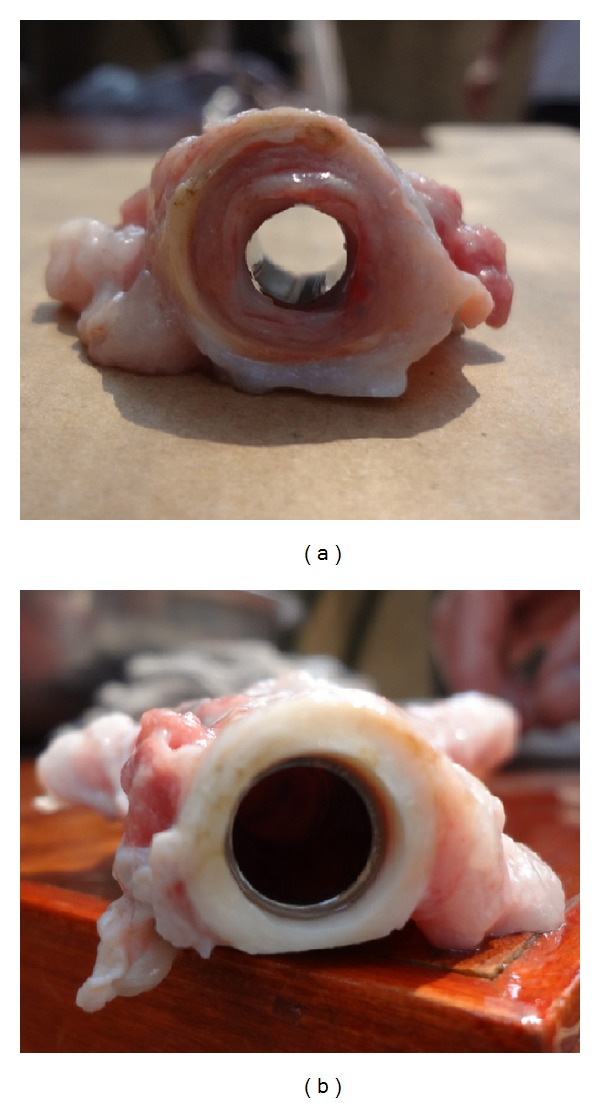
180 days after the operation, the cross-sectional view of the implanted artificial vessel showed that the outer wall was covered by connective tissue and inner wall remained smooth without any thrombus (a); the ligated aortic wall was not necrotic (b).

After sacrifice, the gross autopsy examination showed that the internal organs were normal. The segment of artificial blood vessel had been covered by the connective tissue and metal connectors had been completely wrapped together with the native vessel. The inside wall of the Gore-Tex was covered by layers of intima, while the inside wall of connector was not covered by any cells (Figure 3(a)). And there was no necrosis observed on the ligated native aorta (Figure 3(b)).

### 3.3. Blood Compatibility

**Figure 4:**
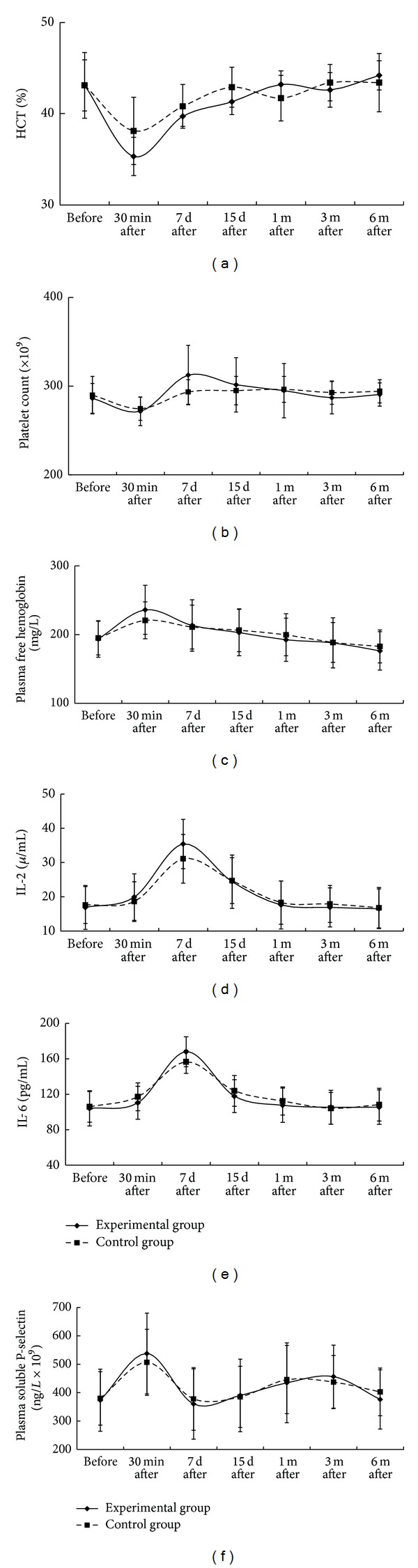
The change curves for each index at each time point. Panels (a), (b), (c), (d), (e), and (f) showed that the changes of the HCT, the platelet count, plasma free hemoglobin, IL-2, IL-6, and the plasma soluble P-selectin, respectively, were not significantly different (*P* > 0.05), when compared between groups at each time point.

The HCT of both groups decreased significantly 30 minutes after the operation and returned to the normal level after 1 month. Comparison of HCT between the groups at each time point showed no significant difference (*P* > 0.05, Figure 4(a)). The platelet count decreased to the lowest level of (262 ± 22) × 10^9^ 30 minutes after the operation, increased to the highest level of (318 ± 46) × 10^9^ 7 days later, and returned slowly to the normal level of (294 ± 42) × 10^9^ to (283 ± 25) × 10^9^ 1 month later. No significant difference was observed when comparing the platelet count of the groups at each time point (*P* > 0.05, Figure 4(b)). Compared with the baseline, the plasma free hemoglobin reached the highest value of (235.98 ± 35.75) mg/L and then fell slightly to the level of (176.18 ± 28.01) mg/L. The changes of plasma free hemoglobin at each time point were not significantly different between groups (*P* > 0.05, Figure 4(c)). As detected by ELISA, IL-2 (Figure 4(d)) and IL-6 (Figure 4(e)) began to increase 30 minutes after the operation, reached their highest levels 7 days later, and then returned slowly to normal ranges. When compared between groups, the change was not significantly different (*P* > 0.05). The plasma soluble P-selectin reached the highest value of (537.82 ± 142.34) ng/L × 10^9^ at postoperation day 1 and significantly decreased to the range of (360.18 ± 123.69) ng/L × 10^9^ to (456.50 ± 110.80) ng/L × 10^9^ 1 month later. When compared between groups, the change trend was not significantly different (*P* > 0.05, Figure 4(f)).

### 3.4. Light Microscopy and Transmission Electron Microscopy

**Figure 5:**
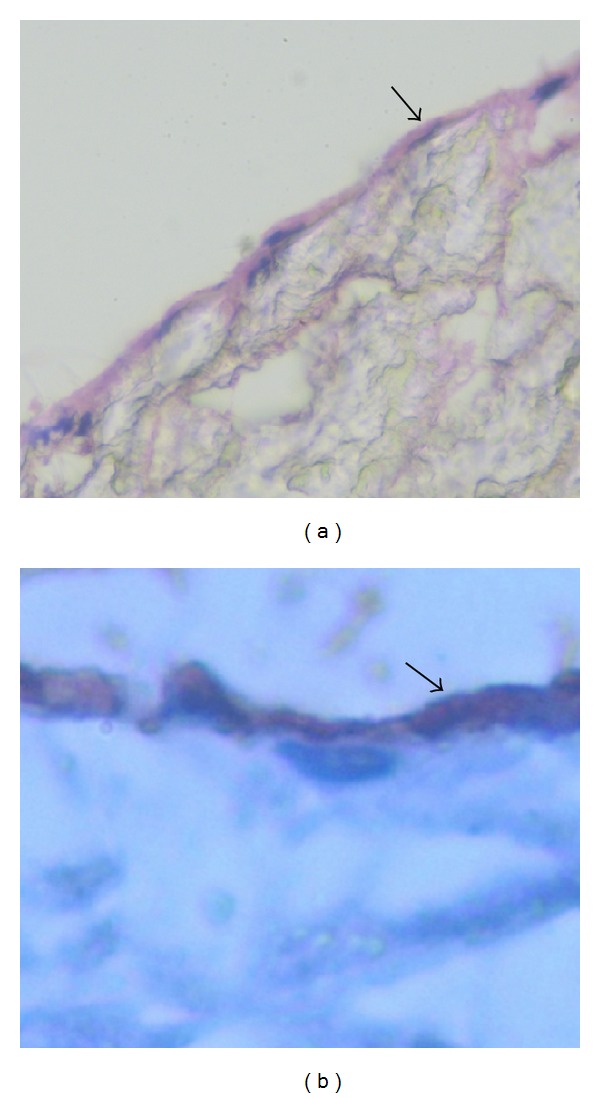
180 days after the operation, endothelial cells were found attached on the surface of the artificial blood vessel wall. Panel (a) H&E and panel (b) CD^34+^ staining (400x).

**Figure 6:**
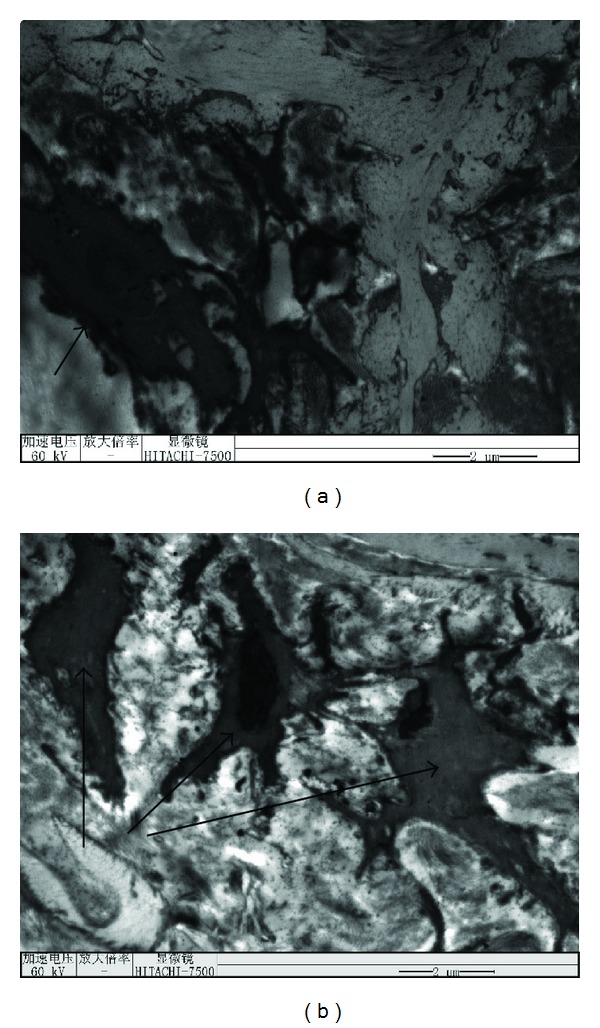
180 days after the operation, endothelial cells were found adhered to the inner wall of the artificial vessels (panel (a), arrow) and smooth muscle cells (panel (b), arrow) without any thrombosis (5000x).

H&E staining showed that there were endothelial cells attached on the inner surface of the artificial vessel (Figure 5(a), arrow), but these endothelial cells did not form a complete membrane covering the inner wall of artificial vessel. The attached endothelial cells were further confirmed by CD^34^ staining (Figure 5(b), arrow). Transmission electron microscopy showed that the inner wall of the SMSBV was attached by vascular endothelial cell (Figure 6(a), arrow) and smooth muscle cells (Figure 6(b), arrow) without any thrombosis.

## 4. Discussion

Great artery diseases such as coarctation of the aorta, aneurysm of thoracic aorta, and aortic dissection are common diseases in cardiovascular surgery, and most of these diseases need surgical replacement with artificial blood vessels. Right now, the most commonly used artificial vascular vessels are made from polytetrafluoroethylene vascular graft [4] and others from decellularization of swine blood vessel [5].

When replacing the aortic lesion with artificial blood vessel, the surgeon must anastomosis the artificial vessel with the native vessel stitch by stitch, and in most cases this handmade anastomosis must be carried under the support of cardiopulmonary bypass and even deep hypothermia cardiopulmonary bypass or circulatory arrest [6]. It is a time-consuming effort to perform a vascular anastomosis by hand and anastomotic bleeding is one of the most common complications that may compromise the surgical outcome for a successful great artery operation [7]. In addition, prolonged cardiopulmonary bypass or circulatory arrest is often associated with serious postoperative complications of the central nervous system [8, 9]. Recently, with the development of the new artificial blood vessel [10, 11], wide applications of noninvasive suture, fibrin glue hemostasis, aprotinin [12], and improvements of extracorporeal perfusion techniques, the mortality for great artery replacement has decreased significantly. But the mortality rate for AD operation remains as high as 9%–23% [13]. The following reasons contribute to the mortality. (1) The vascular anastomosis is technically demanding and prone to causing anastomotic bleeding. This is especially noticeable in cases of acute aortic dissection caused by aortic wall edema, resulting in anastomotic bleeding from the pinholes and is difficult to be controlled. (2) The deep hypothermic circulatory arrest technique has been used widely and its safe duration has been overestimated. It has been reported that the prolonged duration of deep hypothermic circulatory arrest for more than 25 minutes may lead to temporary or permanent postoperative neurological dysfunction. And the supplemented retrograde cerebral perfusion may also increase postoperative transient or permanent neurologic dysfunction [14, 15]. (3) The pulmonary injury caused by cardiopulmonary bypass and/or circulatory arrest as well as the operative blood loss associated with massive blood transfusion may prolong the duration of mechanical ventilation [16], which in turn increases morbidity pulmonary complications. Therefore, simplifying vascular anastomosis technique, shortening the aortic block time, and eliminating the anastomotic bleeding are key points to decrease the mortality and morbidity for thoracic aortic aneurysm surgery. Some scholars have conducted some experimental and clinical studies for using hard-sleeve sutureless artificial aortic vessel [17, 18] and found that the sutureless vessel has the advantages of shortened cardiopulmonary bypass time and/or aortic block time and lower incidence of anastomotic bleeding than conventional handmade suture. But no further clinical studies related to this hard-sleeve artificial aortic vessel have been reported.

In this study, using the SMSBV, the replacement of thoracic descending aorta can be completed within a relative short time period and does not need the support of cardiopulmonary bypass. After the implantation of the SMSBV, the HCT and platelet count decreased, whereas the plasma free hemoglobin and soluble P-selectin increased in the same period. But the changes were not significantly different between groups and returned to normal level 30 days later. In both groups, the IL-2 and IL-6 showed similar changes after the operation. The similar changes of HCT, platelet count, plasma free hemoglobin, P-selectin, and IL-2, IL-6 indicated that the surgical injury and the existence of artificial vessels evoked an early postoperative inflammatory reaction lasting only for a few weeks. Therefore, we conclude that the SMSBV has good blood compatibility. The postoperative biopsy findings showed that the metal connectors and Gore-Tex tube were completely wrapped by connective tissue. The pathological examination and the electron microscopy showed that the inner wall of the artificial vessel wall was covered with layer of endothelial cells and vascular smooth cells without any thrombosis. Pig's intimal proliferation may be much more intense than that in human being [19], which means that the healing capacity may be greater than that in a diseased human aorta, but the difference may not enough to impede we conclude, from these biopsy findings, that the SMSBV has a good biocompatibility.

This SMSBV, with good blood compatibility and biocompatibility, can simplify the anastomoses procedure, shorten operative time, and reduce related complications and did not yield any adverse effects to the experimental animals. So, the SMSBV should be applied wildly even in the era of increasing endovascular activity. Theoretically, our SMSBV can also be used in congenital coarctation of the aorta and aortic pseudoaneurysm, especially in the case when the narrowed aorta is too long and it is impossible to directly anastomose the remaining aorta end to end without using an artificial vessel. In addition, the SMSBV may be used in patients with aortic dissection or pseudoaneurysm whose arterial wall structure shows signs of damage, degeneration, and edema, using two mats to clip the aortic wall and then ligate it the metal connector is recommended. 

## 5. Conclusion

The SMSBV has good biocompatibility and blood compatibility; it can simplify the surgical procedure and shorten the anastomosis time and, thereby, reduce the associated complications such as anastomotic bleeding to improve surgical outcome. Although the application is wide, a large-scale clinical trial should be established to further confirm the safety and risk-to-benefit ratio of this SMSBV.
